# Hyponatremia Secondary to Psychogenic Polydipsia and Schizophrenia: A Case Report

**DOI:** 10.7759/cureus.64600

**Published:** 2024-07-15

**Authors:** Matthew R Nickles, Gagandeep Singh

**Affiliations:** 1 Psychiatry, Edward Via College of Osteopathic Medicine, Blacksburg, USA; 2 Psychiatry, New River Valley Community Services, Blacksburg, USA

**Keywords:** schizophrenia, polydipsia and polyuria, hyponatremia, primary polydipsia, psychogenic polydipsia

## Abstract

Psychogenic polydipsia is characterized by excess thirst, followed by the overconsumption of liquids. This condition is seen in an array of mental illnesses, especially schizophrenia. Psychogenic polydipsia can lead to hyponatremia, which can lead to neurologic sequelae, such as seizures, cerebral edema, and death. In the case under study, the patient presents with schizophrenia, fatigue, weakness, and dizziness during psychiatric follow-up. A comprehensive metabolic panel (CMP) was ordered, which indicated hyponatremia. This patient was treated with salt tablets and behavioral therapy, which led to the normalization of his serum sodium and symptom improvement. The patient has a history of psychogenic polydipsia with hyponatremia, treated on and off for years with salt replacement and water restriction. Management of psychogenic polydipsia is a difficult task. It is important to understand the available management options so that water intoxication and the consequences of hyponatremia do not occur.

## Introduction

Polydipsia refers to excessive fluid intake [1]. Polydipsia progresses through three phases: initially polydipsia and polyuria, followed by hyponatremia, and finally water intoxication [2]. The pathogenesis for polydipsia is unclear but may involve hypersensitivity to vasopressin, increased dopamine activity, and defects in osmoregulation [2]. Multiple medical conditions can lead to polydipsia including, a syndrome of inappropriate antidiuretic hormone, diabetes mellitus, and diabetes insipidus [3]. Psychogenic polydipsia occurs in many mental health conditions, including obsessive-compulsive disorder, bipolar disorder, personality disorders, alcohol addiction, and schizophrenia [4]. Among psychiatric patients, schizophrenia patients are the most likely to develop psychogenic polydipsia. In a cross-sectional survey involving 38 polydipsic psychiatric patients, 80% of them had diagnoses of schizophrenia [4]. Other risk factors for psychogenic polydipsia are smoking and chronicity of schizophrenia [5]. Smoking has been shown to increase the risk of polydipsia in individuals with schizophrenia by 2.5 times compared to schizophrenic patients who do not smoke, which was demonstrated in a retrospective study of 61 chronic inpatient psychiatric patients [6]. Prolonged psychiatric hospitalization, Caucasian race, and male gender also increase the risk of psychogenic polydipsia [7].

Psychogenic polydipsia is important because it can lead to major consequences if left untreated. This includes hyponatremia, which can cause nausea, vomiting, ataxia, delirium, and death [8]. In a review of 27 patients who died before the age of 53 while in a mental hospital, 18.5% died due to complications of water intoxication [9]. In a retrospective study involving 48 individuals suffering from polydipsia and schizophrenia and 42 non-polydipsic schizophrenics, life expectancy was 13% shorter in schizophrenic patients with polydipsia [7].

Management of hyponatremia and psychogenic polydipsia has proven difficult for providers due to the paucity of definitive treatment options. Fluid restriction is the gold standard for this condition but usually fails due to a lack of compliance and strong compulsions to drink liquids because of excess thirst [2]. Many medications have been used to manage this condition, including acetazolamide [10], naltrexone [11,12], olanzapine [13], clozapine [14], and salt replacement [15]. Behavioral therapy is a viable treatment option for psychogenic polydipsia and has been successful in reducing water intake in multiple case reports [16,17]. Therefore, in this brief report, we discuss the case of a 45-year-old male with schizophrenia and psychogenic polydipsia, including possible management strategies and the challenges associated with management.

## Case presentation

This is a case of a 45-year-old, single, white Caucasian male who was seen for psychiatric follow-up. The patient had a medical history of Type 2 diabetes mellitus and hypothyroidism managed with metformin and levothyroxine. His vital signs included a height of 5′ 5″, a weight of 189 lbs, blood pressure of 110/80, a heart rate of 89 beats per minute, and a body mass index of 31.45. At the time of presentation, he reported smoking two packs of cigarettes per day and drinking three gallons of water per day.

The patient’s psychiatric history began in early childhood, but he was diagnosed with schizophrenia in his mid-twenties. His symptoms of schizophrenia included delusions, such as having 300 children, with two of them being US presidents. He also spoke about many business deals where he received a large sum of money. The patient was experiencing auditory hallucinations, but tolerating them well. His schizophrenia was being managed with clozapine due to refractoriness to previously prescribed antipsychotics. At the time of presentation, the patient was stable on this medication and he had been compliant with obtaining complete blood counts to determine his absolute neutrophil count. This patient was also seeing a case manager two times per week and was a part of the assertive community treatment (ACT) program, where he received supportive services.

During psychiatric follow-up for paranoid schizophrenia, the patient’s primary concern was his symptoms of dizziness, fatigue, and weakness. The patient had a long history of compulsive water drinking that previously resulted in hyponatremic states. In the past few years, he had comprehensive metabolic panels (CMPs) to identify low serum sodium levels, as indicated in Tables 1-2. The patient’s psychogenic polydipsia had been managed in the past by his primary care physician with the use of salt tablets, which increased his serum sodium levels and improved his clinical presentation. For multiple years, the patient had been on and off salt tablets to address his low sodium level. After discontinuing the salt tablets when his sodium levels normalized, his compulsive water drinking continued, leading back to hyponatremia again. The patient had also attempted water restriction and behavioral therapy without long-term success. The patient’s mother stated that she was worried his sodium levels could be low again due to the symptoms he was having. The patient did not see a problem with his water consumption and stated that he only drinks until he is not thirsty anymore. The patient was referred to his primary care physician for management of this condition and will obtain a CMP at his next visit to check serum sodium levels. If hyponatremic, the patient will continue to be managed with salt tablets and water restriction to correct his sodium levels. His symptoms will be monitored in future appointments, and he will complete more CMPs to watch his sodium levels.

**Table 1:** CMP results in May 2021 Na: sodium; K: potassium; Cl: chloride; RBC: red blood cell; Hgb: hemoglobin; Hct: hematocrit; MCV: mean corpuscular volume; CMP: comprehensive metabolic panel

Investigation	Patient value	Normal range
Na	126 mmol/L	135-146 mmol/L
K	3.4 mmol/L	3.5-5.3 mmol/L
Cl	89 mmol/L	98-110 mmol/L
RBC	3.92 million/µL	4.2-5.8 million/µL
Hgb	12.5 g/dL	13.2-17.1 g/dL
Hct	36.60%	38.5-50%
MCV	93.4 fL	77-98 fL
Calculated osmolality	255 mos/kg	278-305 mos/kg

**Table 2:** CMP results in February 2022 Na: sodium; K: potassium; Cl: chloride; RBC: red blood cell; Hgb: hemoglobin; Hct: hematocrit; MCV: mean corpuscular volume; CMP: comprehensive metabolic panel

Investigation	Patient value	Normal range
Na	125 mmol/L	135-146 mmol/L
K	3.5 mmol/L	3.5-5.3 mmol/L
Cl	92 mmol/L	98-110 mmol/L
RBC	3.87 million/µL	4.2-5.8 million/µL
Hgb	12.3 g/dL	13.2-17.1 g/dL
Hct	36.00%	38.5-50%
MCV	93.0 fL	77-98 fL
Calculated osmolality	255 mos/kg	278-305 mos/kg

## Discussion

Psychogenic polydipsia is a potentially fatal condition that can lead to life-threatening consequences, such as seizures, cerebral edema, and rhabdomyolysis [18]. In this case, the patient was experiencing minor symptoms of hyponatremia, such as fatigue and dizziness, which often precede more dangerous complications. Psychogenic polydipsia is a common condition occurring in many psychiatric conditions, with the most common being schizophrenia [4]. The patient in this report has a diagnosis of paranoid schizophrenia, which has been managed pharmacologically since his mid-twenties. Many risk factors for psychogenic polydipsia are present in this patient, including schizophrenia [4], smoking cigarettes [6], and being a Caucasian male [7]. His history of smoking, combined with schizophrenia, more than doubles his chance of having psychogenic polydipsia [6].

There are various ways to manage psychogenic polydipsia, but none of them are definitive. In a case report involving five patients with psychogenic polydipsia, four of the patients saw improvement in their condition after treatment with acetazolamide, which is a carbonic anhydrase inhibitor [10]. In an open-design study, naltrexone was used to manage psychogenic polydipsia in seven schizophrenic patients. After 6 weeks, there was an improvement in diurnal weight gain and a reduction in polydipsic mannerisms [11]. In a more recent case report, a patient suffering from psychogenic polydipsia was successfully managed with a combination of naltrexone and irbesartan [12]. In a case report involving a patient with paranoid schizophrenia and severe polydipsia, atypical antipsychotics were used to manage the behavior. Risperidone improved polydipsic behavior for a short period but could not sustain it, while olanzapine improved behavior after the addition of irbesartan [13]. Clozapine demonstrated the ability to decrease compulsive drinking behavior in a 68-year-old female in acute psychosis, improving the patient’s sodium from 113 mmol/L to 143 mmol/L [14].

Besides pharmacological therapy, behavioral therapy can be used to manage psychogenic polydipsia. In a case report, a patient was able to reduce his liquid intake by using two different methods. The first method included only allowing the patient to drink water through a narrow straw, making it more difficult to over-consume, and pairing drinking with a low-preference activity. The second method was positively reinforcing water refusal with rewards, such as candy and gum while pairing drinking with a no-activity [16]. Stimulus control, relaxation training to help with distress associated with the urge to drink water and response prevention have also been successful in reducing water intake [17].

For the patient under study, the major problem continues to be his behavior of compulsive water drinking triggered by intense urges to consume fluids. Salt replacement has shown to be an effective management option to normalize the patient’s sodium concentration and improve his symptoms of hyponatremia, but without a change in his behavior, his hyponatremia continues to recur. Attempting the use of a different medication or behavioral therapy may be beneficial in correcting his compulsive water-drinking behavior, which in turn, would lead to better health outcomes.

Moreover, routine assessment of water intake in patients with schizophrenia is important so that psychogenic polydipsia and hyponatremia can be diagnosed promptly and managed to prevent adverse outcomes.

## Conclusions

Psychogenic polydipsia is a dangerous condition that can lead to hyponatremia and cause severe consequences. It is important to screen patients with mental illness and those with signs of hyponatremia to manage their condition. Although behavioral therapy, water restriction, medications, and salt replacement are beneficial to patients with psychogenic polydipsia, more research, large-scale studies, and clinical trials are needed to create a treatment guideline.
